# Case Report: A heterozygous mutation of NLRP3 in a Chinese child with NLRP3-AID

**DOI:** 10.3389/fped.2025.1411603

**Published:** 2025-08-07

**Authors:** Yangming Ruan, Ting Ge, Yizhong Wang, Ting Zhang, Feifei Song

**Affiliations:** Department of Gastroenterology, Hepatology, and Nutrition, Shanghai Children’s Hospital, Shanghai Jiao Tong University, Shanghai, China

**Keywords:** NLRP3-associated autoinflammatory disease (NLRP3-AID), neonatal-onset multisystem inflammatory disorder (NOMID), NLRP3 gene, cryopyrin-associated periodic syndrome (CAPS), case report

## Abstract

**Background:**

NLR family pyrin domain containing 3 (NLRP3)–associated autoinflammatory disease (NLRP3-AID), formerly known as cryopyrin-associated periodic syndrome, is a group of AIDs comprising neonatal-onset multisystem inflammatory disorder, Muckle–Wells syndrome, and familial cold autoinflammatory syndrome. Mutations in the NLRP3 gene are considered central to its pathogenesis.

**Case report:**

Here, we present a Chinese infant diagnosed with severe NLRP3-AID who carried a heterozygous variant in the NLRP3 gene. The patient exhibited recurrent episodes of fever, urticaria-like rashes, aseptic meningitis, and hearing loss. During hospitalization, elevated inflammatory markers and leukocytosis in body fluids were observed without evidence of infection. DNA sequencing identified a *de novo* heterozygous mutation, c.1006A > G (p.I336V), in the NLRP3 gene.

**Conclusion:**

We report an infant with NLRP3-AID and emphasize the importance of early diagnosis based on clinical manifestations.

## 1 Introduction

NLR family pyrin domain containing 3 (NLRP3)–associated autoinflammatory disease (NLRP3-AID) is an autoinflammatory disease that was once considered to be three distinct clinical entities: neonatal-onset multisystem inflammatory disorder (NOMID)/chronic infantile neurologic, cutaneous, and articular (CINCA); Muckle–Wells syndrome (MWS); and familial cold autoinflammatory syndrome (FCAS) (1). However, in 2001, gain-of-function mutations in the NLRP3 gene (1q44) were identified as the cause of these three disorders, and NLRP3-AID was subsequently classified as a syndrome with varying disease severity (3).

NOMID/CINCA presents the most severe phenotype, featuring persistent neonatal-onset inflammation that affects the nervous system (aseptic meningitis, cognitive impairment), joints (cartilage overgrowth/deformity), skin (chronic urticarial rash), and eyes (uveitis). MWS presents with recurrent fever, urticaria, and progressive sensorineural hearing loss. It is also associated with long-term complications such as renal amyloidosis. FCAS, the mildest form, is characterized by self-limiting episodes triggered by cold exposure, typically involving fever, urticaria, and arthralgia (2–6). Notably, the boundaries between these three syndromes are not always clear.

The broad but non-specific manifestations, coupled with the rarity of NLRP3-AID, contribute to diagnostic challenges. In China, patients with NLRP3-AID are rarely reported, and clinicians often lack familiarity with the disease, resulting in delayed or incorrect diagnoses (7). Here, we present an infant with severe NLRP3-AID who carried a heterozygous NLRP3 mutation, which is novel in China, and compare this case with cases involving the same mutation reported in Japan and France. We emphasize the importance of recognizing NLRP3-AID based on clinical manifestations, particularly in cases of recurrent aseptic meningitis, fever, and rashes, beyond relying solely on genetic testing.

## 2 Case report

A 4-month-old female infant was admitted to our hospital with a 5-day febrile illness without other symptoms. The patient was born at full term, and her family history was unremarkable. Blood tests showed leukocytosis with neutrophilic predominance (56.4%) and elevated levels of C-reactive protein (CRP; 90 mg/L), serum amyloid A (SAA, 81.99 mg/L), erythrocyte sedimentation rate (48 mm/h), procalcitonin (1.65 ng/mL), and platelet count (442 × 10^9^/L) (Table 1). The patient’s immunoglobulin levels and a lymphocyte subset analysis were normal. The tests for autoantibodies, including the anti-dsRNA antibody, were negative (Supplementary Table S1). The urinalysis revealed a white blood cell (WBC) count of 55/µL and erythrocyte count of 31.1/µL. The cerebrospinal fluid (CSF) examination also showed elevated white blood cell counts, increased protein levels, and decreased glucose levels. Extensive microbiological investigations of the CSF, including next-generation sequencing (NGS), found no evidence of infection. Stool analysis and renal and liver function tests were normal, with no cough, frequent urination, seizures, or other remarkable symptoms. A fundoscopic examination and brain magnetic resonance imaging showed no abnormalities.

**Table 1:** Initial laboratory findings at admission: hematological and inflammatory parameters.

Blood index	Count	Ratio (%)	Reference range
White blood cell (WBC, ×10^9^/L)	14.68		8.00–12.00
Platelet (PLT, ×10^9^/L)	442		100–400
Hemoglobin (Hb, g/L)	94		110–160
C-reactive protein (CRP, mg/L)	90		0–5
Serum amyloid A (SAA, mg/L)	81.99		0–10.00
Erythrocyte sedimentation rate (ESR, mm/h)	48		0–20
Procalcitonin (PCT, ng/mL)	1.65		0.00–0.10
Neutrophil (N)	8.28	56.4	2.40–4.80; 30.0–40.0
Lymphocyte (L)	4.95	33.7	4.00–8.40; 50.0–70.0

Laboratory tests showed high-level inflammatory signs in the patient's body fluids.

Urticaria-like rashes appeared 8 days after her admission. Over a 2-month period, multiple antibiotics (meropenem, vancomycin, and ceftriaxone) were administered for suspected urinary tract and central nervous system (CNS) infections. No clinical or biochemical improvement occurred. The patient's body temperature fluctuated daily and CSF examinations consistently showed elevated white blood cell counts (24–336 × 10^6^/L) and protein levels (470–990 mg/L), and low glucose levels (1.9–2.6 mmol/L). These findings contrasted with her relatively mild presentation, with no CNS symptoms. The patient was finally discharged after partial symptom improvement, with the CSF indicators showing some improvement. In addition, the patient did not pass the otoacoustic emissions test in her left ear during hospitalization. Retrospectively, a similar episode had occurred during the neonatal period, characterized by aseptic meningitis and urticaria-like rashes.

Long-term antibiotic treatment with no obvious signs of improvement, along with early and recurring meningitis episodes, prompted consideration of diseases associated with immune dysregulation, and NGS was performed.

**Figure 1:**
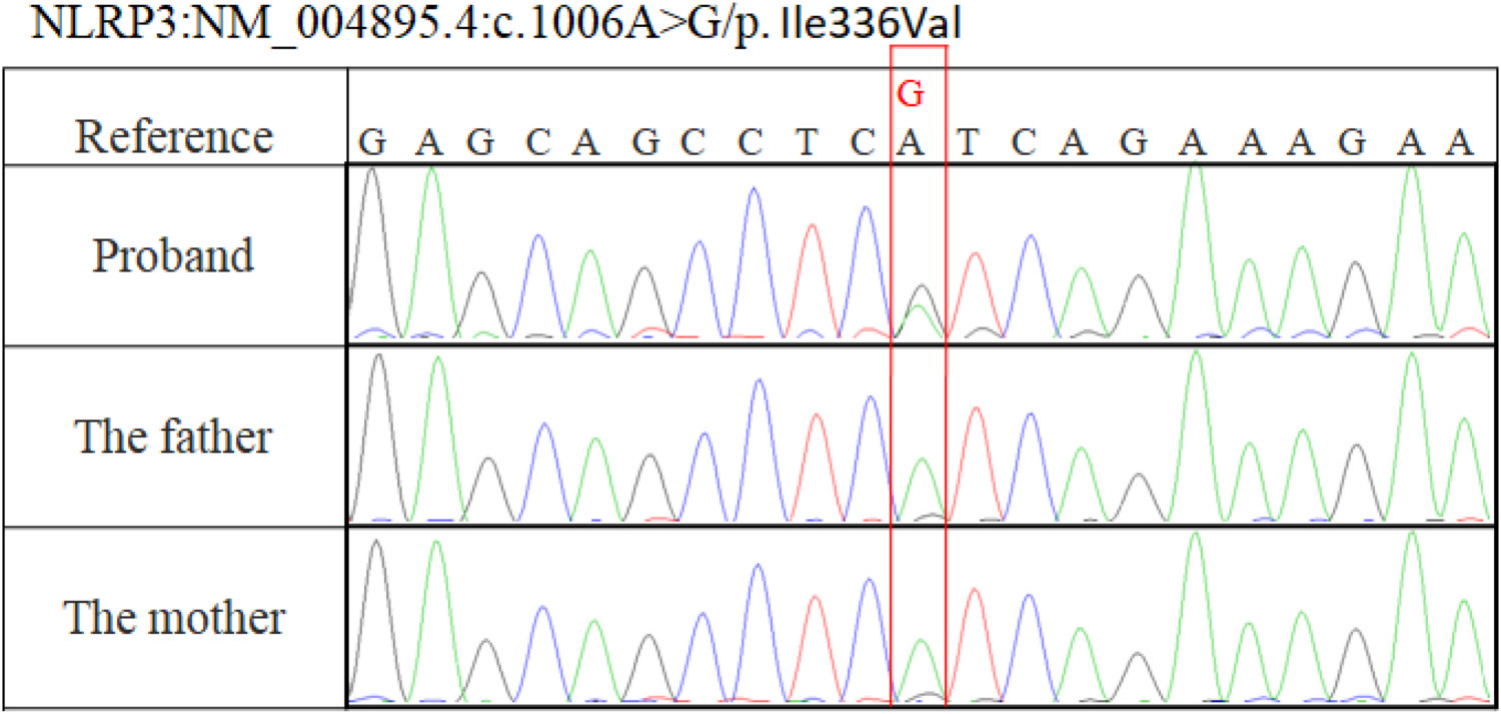
Sanger sequencing showed a c.1006A>G (p.Ile336Val) variation in our patient and no mutation in her parents.

Whole blood ethylenediaminetetraacetic acid (EDTA) samples from the patient and her parents were used to extract genomic DNA according to established protocols using a QIAamp DNA Blood Mini Kit (Qiagen, Hilden, Germany). Exome capture was performed using the IDTxGen® Exome Capture Kit (IDT, Coralville, IA, USA) and sequenced on HiSeq X10 (Illumina, San Diego, CA, USA). The sequencing data achieved 99.31% coverage at ≥50×. A heterozygous c.1006A>G (p.Ile336Val) *de novo* mutation in the NLRP3 gene was detected and confirmed by Sanger sequencing (Figure 1).

Based on the sequencing results and the presence of unprovoked generalized inflammation, including recurrent fever, urticaria-like rashes, aseptic meningitis, and sensorineural hearing loss, NLRP3-AID was determined to be the most likely diagnosis.

The patient was referred to the hematology department but her parents declined further clinical interventions, including hematopoietic stem cell transplantation. The child was followed up monthly in our outpatient clinic for routine blood counts (WBC 13.81–20.98 × 10^9^/L) until 1 year of age, after which she was lost to follow-up. At the age of 3, she returned as an outpatient with upper respiratory tract infection-like symptoms. Her parents reported that an interleukin (IL)-1β blocker (canakinumab) had been administered by an external hospital 8 months after the onset of her illness. Since starting the medication, the patient had not experienced similar episodes but continued to exhibit mild sensorineural hearing loss. A follow-up blood test 2 months later showed normal WBC (9.73 × 10^9^/L) and CRP levels (<5 mg/L) (Supplementary Table S2).

## 3 Discussion

AIDs are a group of disorders associated with innate immune dysfunction, receiving increasing attention since the 1990s (8–10). The broad symptom spectrum of AIDs has led to multiple definitions. In 2018, a consensus defined AIDs as clinical disorders characterized by recurrent or continuous inflammation without the pathogenic involvement of the adaptive immune system, caused by defects or dysregulation of the innate immune system (11). To date, a total of 51 genes associated with 55 autoinflammatory conditions have been identified in the Infevers database (https://infevers.umai-montpellier.fr). According to the consensus, the proposed nomenclature for cryopyrin-associated periodic syndrome (CAPS) was subsequently revised to NLRP3-associated AID based on a new understanding of the NLRP3 gene (11). NLRP3-AID can be classified into three levels of severity: severe, moderate, and mild.

NLRP3-AID represents a family of diseases that share a common etiology of gain-of-function mutations in the NLRP3 gene, most crucially resulting in elevated IL-1 production. The NLRP3 gene encodes a core component of the NLRP3 inflammasome that activates procaspase-1, converting it to active caspase-1. Caspase-1 then cleaves pro-inflammatory cytokines, including IL-1β and IL-18 (1). IL-1β induces a cascade of downstream signals, leading to the activation of nuclear factor kappa B (NF-κB) and the release of other inflammatory cytokines. It is a major mediator of fever in humans (12). Overactivation of the NLRP3 inflammasome can cause systemic inflammation, including recurrent fever, urticaria-like rashes (with or without cold exposure), arthritis, aseptic meningitis, hearing loss, mental and growth retardation, and bony overgrowth (13). The incidence rate of NLRP3-AID is estimated to be approximately 1–2 per 1,000,000 in the United States and approximately 1 per 360,000 in France. No incidence data are available for China (14).

Most of the reported pathogenic mutations are located in the NACHT domain (5, 15), which has ATPase activity and is essential for NLRP3 self-association and function (16). The c.1006A>G (p.Ile336Val) mutation identified in our case is also located in the NACHT domain, supporting its pathogenicity. Due to different transcript references among laboratories, an I336V mutation can also be designated I334V. This mutation has been previously reported in two individuals (in 2013 and 2017) and was associated with MWS and NOMID, respectively (17, 18). Based on the clinical presentation, laboratory findings, and sequencing results of our patient—particularly her neonatal onset of aseptic meningitis and urticaria-like rashes without specific cold exposure—severe NLRP3-AID (NOMID) was considered the most appropriate diagnosis.

All three reported cases share common features, including recurrent fever, rashes, and hearing loss. However, unlike the previously reported cases, our patient presented with aseptic meningitis at onset, with no ocular or joint involvement (Table 2). Interestingly, despite the early and recurrent development of aseptic meningitis, our patient did not display typical signs of intracranial hypertension, such as vomiting or papilledema. In addition, laboratory tests revealed an increased white blood cell count in urine, consistent with systemic inflammation caused by the NLRP3 mutation. This finding was not observed in the other reported cases and is rarely mentioned in other variant reports (19). This observation may broaden the clinical manifestation spectrum of NLRP3-AID. The varied manifestations and severity of the same mutation in different patients align with previous studies (20), supporting the hypothesis that the NLRP3 mutation alone cannot fully elucidate disease etiology (1, 14). Other factors, such as epigenetic modifications, may contribute to the pathogenesis (21).

**Table 2:** Clinical features comparison in patients with the c.1006A > G (p.Ile336Val) mutation within the NLRP3 gene.

Patient no.	Nation	Reference	Age at onset	Symptoms at onset	Systemic involvement			
					Neurology	Eye	Joint	Urinary tract
1	Japan	Nakagawa et al. (17)	2 years	Arthralgias	Aseptic meningitis and headache	None	Arthritis	None
2	France	Papa et al. (18)	At birth	NA	Aseptic meningitis, headache, vomiting, and papilledema	Uveitis anterior and impaired vision	Arthritis	None
3	China	Present study	At birth	Aseptic meningitis	Aseptic meningitis	None	None	Pyuria

NA, not available.

Our patient presented with aseptic meningitis with milder clinical manifestations. She did not show ocular or joint involvement and she had pyuria.

The rarity of this disease leads to fragmented clinical information, with limited experience available from only a few specialized centers. The complex molecular pathogenesis and potential environmental and epigenetic factors further complicate our understanding of the disease. Therefore, a delayed diagnosis is not uncommon due to the rarity and phenotypic variability of NLRP3-AID (7, 22). Genetic analysis, especially with the advent of NGS, has significantly supported the diagnosis of NLRP3-AID (5), as demonstrated in our case. However, data suggest that increasing reliance on genetic analysis does not always improve detection and may sometimes lead to overuse due to clinicians’ unfamiliarity with this rare disease (19). The 2017 expert consensus excluded genetic evidence from NLRP3-AID diagnostic criteria. These diagnostic criteria performed equally well in mutation-positive and mutation-negative patients, showing a sensitivity of 81% and specificity of 94% (22). Diagnosis is based on raised inflammatory markers and the presence of at least two of six clinical features: urticaria-like rash, cold-triggered episodes, hearing loss, musculoskeletal symptoms, chronic aseptic meningitis, and epiphyseal overgrowth. Our patient exhibited elevated inflammatory markers (CRP and SAA), urticaria-like rash, sensorineural hearing loss, and chronic aseptic meningitis, meeting the diagnostic criteria for early diagnosis. Accurate genetic diagnosis remains important, not only for confirmation but also for facilitating personalized care (23).

Treatment of NLRP3-AID focuses on suppressing IL-1β using IL-1β cascade inhibitors, including canakinumab, anakinra, and rilonacept, which have shown promising results (24). However, IL-1 blockers have not yet completed clinical trials in China (14), and our patient received treatment from an external hospital.

## 4 Conclusion

We reported a Chinese female infant with NLRP3-AID caused by a heterozygous mutation in the NLRP3 gene. This variant was reported in a Chinese patient for the first time. We recommend considering NLRP3-AID in infants with recurrent fever, rash, and aseptic meningitis. This approach facilitates earlier diagnosis prior to genetic confirmation.

## Data Availability

The raw data supporting the conclusions of this article will be made available by the authors, without undue reservation.
