# Supplementary material for: Case Report: A heterozygous mutation of NLRP3 in a Chinese child with NLRP3-AID
Source: Front Pediatr. 2025 Aug 7;13:1411603. doi: 10.3389/fped.2025.1411603 (PMC12369556; doi:10.3389/fped.2025.1411603)
Supplement: Supplementary file 1 [file Table1.docx]

**Supplementary Tables**

**Supplementary Table 1.**

Antibody identification panel.

| Autoantibodies | Result |
| --- | --- |
| Anti-dsRNA antibody | − |
| Anti-ribonucleoprotein antibody | − |
| Anti-SM antibody | − |
| Anti-sicca syndrome A antibody | − |
| Anti-sicca syndrome B antibody | − |
| Anti-ScL-70 antibody | − |
| Anti-centromere protein B antibody | − |
| Anti-Jo-1 antibody | − |
| Anti-nucleosome antibody | − |
| Anti-histone antibody | − |
| Anti-ribosomal P protein antibody | − |
| Anti-proliferation cell nuclear antigen antibody | − |
| Anti-PM-ScL 100 antibody | − |
| Anti-mitochondrial antibody | − |

PM, polymyositis; ScL, sclerosis; −, negative

The patient showed no evidence of autoimmune disease.

**Supplementary Table 2.**

Hematologic and inflammatory parameters after treatment.

| Blood index | Count | Ratio (%) | Reference range |
| --- | --- | --- | --- |
| White blood cell (WBC; ×10^9^/L) | 9.73 |  | 4.00–10.00 |
| Platelet (PLT; ×10^9^/L) | 183 |  | 100–400 |
| Hemoglobin (Hb; g/L) | 127 |  | 110–160 |
| C-reactive protein (CRP; mg/L) | <5.00 |  | 0.00–5.00 |
| Neutrophil (N) | 3.42 | 35.1 | 2.00–7.00; 50.0–70.0 |
| Lymphocyte (L) | 5.28 | 54.3 | 4.00–8.40; 30.0–40.0 |

Laboratory tests showed normal inflammatory signs in patient’s complete blood count after treatment.
